# Molecular targets and signaling pathways regulated by nuclear translocation of syndecan-1

**DOI:** 10.1186/s12860-017-0150-z

**Published:** 2017-12-08

**Authors:** Tünde Szatmári, Filip Mundt, Ashish Kumar-Singh, Lena Möbus, Rita Ötvös, Anders Hjerpe, Katalin Dobra

**Affiliations:** 1 Department of Laboratory Medicine, Division of Pathology, Karolinska Institutet, SE-14186 Stockholm, Sweden; 2 Division of Clinical Pathology/Cytology, Karolinska University Laboratory, Karolinska University Hospital, SE-14186 Stockholm, Sweden

**Keywords:** Syndecan-1, Nuclear localization, Transcriptomic, Proteomic, Pathway analysis, Proliferation, Cell cycle

## Abstract

**Background:**

The cell-surface heparan sulfate proteoglycan syndecan-1 is important for tumor cell proliferation, migration, and cell cycle regulation in a broad spectrum of malignancies. Syndecan-1, however, also translocates to the cell nucleus, where it might regulate various molecular functions.

**Results:**

We used a fibrosarcoma model to dissect the functions of syndecan-1 related to the nucleus and separate them from functions related to the cell-surface. Nuclear translocation of syndecan-1 hampered the proliferation of fibrosarcoma cells compared to the mutant lacking nuclear localization signal. The growth inhibitory effect of nuclear syndecan-1 was accompanied by significant accumulation of cells in the G0/G1 phase, which indicated a possible G1/S phase arrest.

We implemented multiple, unsupervised global transcriptome and proteome profiling approaches and combined them with functional assays to disclose the molecular mechanisms that governed nuclear translocation and its related functions. We identified genes and pathways related to the nuclear compartment with network enrichment analysis of the transcriptome and proteome. The TGF-β pathway was activated by nuclear syndecan-1, and three genes were significantly altered with the deletion of nuclear localization signal: EGR-1 (early growth response 1), NEK11 (never-in-mitosis gene a-related kinase 11), and DOCK8 (dedicator of cytokinesis 8). These candidate genes were coupled to growth and cell-cycle regulation. Nuclear translocation of syndecan-1 influenced the activity of several other transcription factors, including E2F, NFκβ, and OCT-1. The transcripts and proteins affected by syndecan-1 showed a striking overlap in their corresponding biological processes. These processes were dominated by protein phosphorylation and post-translation modifications, indicative of alterations in intracellular signaling. In addition, we identified molecules involved in the known functions of syndecan-1, including extracellular matrix organization and transmembrane transport.

**Conclusion:**

Collectively, abrogation of nuclear translocation of syndecan-1 resulted in a set of changes clustering in distinct patterns, which highlighted the functional importance of nuclear syndecan-1 in hampering cell proliferation and the cell cycle. This study emphasizes the importance of the localization of syndecan-1 when considering its effects on tumor cell fate.

**Electronic supplementary material:**

The online version of this article (10.1186/s12860-017-0150-z) contains supplementary material, which is available to authorized users.

## Background

Syndecan-1 is a transmembrane heparan sulfate proteoglycan (HSPG), which carries heparan-sulfate (HS) and chondroitin-sulfate glycosaminoglycans on its ectodomain. Syndecan-1 acts as a co-receptor for growth factors, chemokines, and cytokines; thus, it regulates a multitude of cellular functions, including cell growth, proliferation, adhesion, and migration [1]. In these processes, the sub-cellular localization of syndecan-1 is critical [2]. Syndecan-1 is typically referred to as a cell-surface proteoglycan, but it can also be found in the stroma [3], and it can be shed into body fluids [4–6]. We have previously reported that syndecan-1 also translocates to the nucleus in a highly regulated manner by a tubulin-mediated transport mechanism [7]. In the nucleus, it co-localizes with FGF-2 and heparanase [8]. Although syndecan-1 has been detected in the nuclear compartment of various tumor types [7, 9, 10], the functions associated with nuclear translocation remain incompletely understood (for review, see [11–13]).

The presence and functions of HS in the nucleus have been studied extensively; however, less research has investigated the translocation of the core protein itself. The nuclear occurrence of HS [14, 15] was lately extended to include the whole syndecan-1 core protein [7, 16]. Other HSPGs, including syndecans-2 and -3 and glypican-1, were also identified in the nuclear compartments of various cell-types [17, 18]. The structural requirement for the nuclear HSPG translocation implies a nuclear localization signals (NLSs) found in the core proteins of several HSPGs. Syndecan-1 harbors the RMKKK motif in the juxta-membrane region of the cytoplasmic domain, which is the minimal, sufficient sequence required for nuclear localization [8]. Moreover, the MKKK sequence is essential for lipid raft-mediated endocytosis [19].

The nuclear HS has an anti-proliferative effect [15, 20], and the extent of growth inhibition depends on the cell confluence, the fine structure and the sulfation pattern of the nuclear HS. Moreover, the effect of nuclear HS differs between malignant and benign cells. The nuclear entry of HS depends on certain cell-cycle phases, and cell cycle progression is regulated by the amount of HS or HSPG in the nucleus [7, 21–25]. However, the exact mechanisms of action have not been established. Another well-studied function of HS is to shuttle heparin-binding growth factors and other macromolecules into the nucleus. These factors are internalized with HSPGs and they co-localize in the nucleus [8, 26–30].

Nuclear HS regulates gene expression through at least two mechanisms. First, it regulates the transcription machinery by inhibiting DNA topoisomerase; this activity prevents DNA relaxation, and the DNA remains inaccessible to transcription factors [31]. Moreover, HS directly inhibits transcription factors [32, 33], probably through direct interactions, because the DNA binding domains of some transcription factors contain high affinity heparin binding sequences [13]. Nuclear HS can also regulate gene expression by modulating the acetylation status of histone proteins. Both nuclear syndecan-1 [34] and HS chains [35] inhibit histone acetyltransferases. This activity can at least partly explain the anti-proliferative effects of HS.

Previously, we stably transfected fibrosarcoma cells with full-length syndecan-1 (FLs1) and a mutated syndecan-1 that lacked the RMKKK nuclear localization signal (NLSdel) motif in the juxtamembrane region of the cytoplasmic domain. We showed that FLs1 entered the nucleus normally, but deletion of the RMKKK motif abolished the nuclear translocation of this proteoglycan [25].

In the current study, we elucidated the functions of nuclear syndecan-1 on both transcriptomic and proteomic levels, combining the results to visualize the affected signaling patterns. With the same two fibrosarcoma cell-sub-lines (one transfected with FLs1 and the other with NLSdel), it was possible to separate the nuclear and cell-surface functions of syndecan-1. We demonstrated a differential impact of nuclear syndecan-1 on cell cycle progression, viability and apoptosis. The transcript of FLs1, translocating to the nucleus (but not the NLSdel mutant, with predominant membrane and cytosolic distribution), induced the accumulation of cells in G1/G0 phase and hampered the proliferation of fibrosarcoma cells. We delineated the molecular background of these changes, and we identified nuclear proteins and transcription factors responsible for these effects.

## Results

### Syndecan-1 level and its subcellular localization in different constructs

Syndecan-1 levels corresponded to 1.5- to 2-fold increase in the FLs1 and NLSdel transfected cell lines compared to controls (Additional file 1: Figure S1).

The subcellular localization of syndecan-1 was confined to the nuclear compartment in cells transfected with FLs1 and it was mainly cytoplasmic in the NLSdel and empty vector transfected cells (Additional file 2: Figure S2).

### Effects of nuclear syndecan-1 on cell proliferation and cell cycle progression

**Fig. 1:**
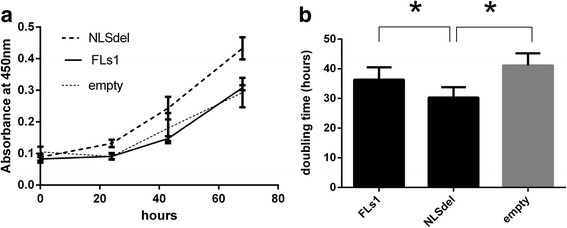
Nuclear translocation of syndecan-1 inhibits fibrosarcoma cell proliferation. **a** B6FS fibrosarcoma cells transfected with full-length (FLs1), syndecan-1 lacking the nuclear localization signal (NLSdel) and corresponding empty vector (empty) were seeded at a density of 3000 cells/well in 96 well plates and absorbance values were measured using WST-1 proliferation assay. Symbols represent the mean ± SD (*n* = 3). Cell proliferation increased when the nuclear translocation of syndecan-1 was impaired in the NLS deleted construct (*p* = 0.018). **b** Doubling time of cells transfected with different constructs shows that NLSdel grows faster while the difference between FLs1 and control is not significant. Bars represent the average of three independent experiments ±SD. *p < 0.05, based on the paired t-test

Proliferation was significantly altered in fibrosarcoma cells transfected with different syndecan-1 constructs. The doubling time of cells transfected with NLSdel was shorter (32.3 h) compared to cells that overexpressed FLs1 (38.9 h) and empty vector (41.09 h); Fig. 1). Consequently, cells with preserved nuclear localization (FLs1) had lower proliferation rate compared to the NLSdel mutant that displayed impaired nuclear localization.

**Fig. 2:**
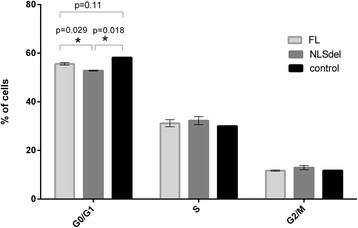
Nuclear translocation of syndecan-1 affects the cell cycle distribution of fibrosarcoma cells. Cells transfected with the full-length (FLs1) or syndecan-1 lacking the nuclear localization signal (NLSdel) and empty vector (empty) were assayed with propidium iodide staining followed by flow cytometry, at 48 h after cell seeding. Columns represent the mean percentage ± SD (n = 3) of cells in the indicated phase of the cell cycle. Cells accumulated in the G1 phase in the presence of nuclear syndecan-1 compared to the deletion mutant. *p values were calculated using the paired t-test

Cell cycle analysis showed that significantly (*p* ≤ 0.05) fewer cells were in the G1 phase in NLSdel cells than in FLs1 cells; thus, cells transfected with NLSdel passed through the G1/S checkpoint more rapidly than cells transfected with FLs1 (Fig. 2).

Immunocytochemical stating with Ki-67 revealed very high proliferation index at 48 h after seeding, corresponding to 99% in all cell lines transfected with the three different constructs. The proportion of Ki-67 positive cells was 83% in empty vector, 94% and 96% in the full-length (FLs1) and NLSdel, respectively, after 72 h (Additional file 3: Figure S3)**.**

### Effects of nuclear translocation of syndecan-1 on the spontaneous apoptosis of fibrosarcoma cells

**Fig. 3:**
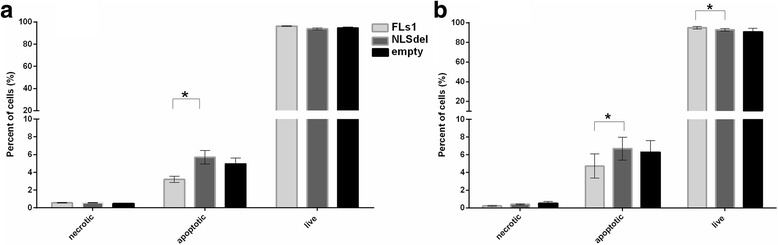
Nuclear translocation of syndecan-1 affects the rate of apoptotic cells in fibrosarcoma cells. Histogram representing flow cytometry data of cells stained with Annexin-FITC and propidium iodide. The percentages of live, necrotic and apoptotic cells were measured at (**a**) 48 h and (**b**) 72 h after cell seeding. The fraction of apoptotic cells was significantly lower in cells transfected with full-length syndecan-1 (FLs1), at both time points, compared to cells transfected with syndecan-1 lacking the nuclear localization signal (NLSdel). Bars represent the average of three independent experiments ± SD. *P < 0.05, based on the paired t-test

Nuclear translocation of syndecan-1 caused a small, but significant (*p* ≤ 0.05) inhibition of spontaneous apoptosis at 48 and 72 h after cell seeding, compared to the apoptosis of cells transfected with NLSdel. Apoptosis of fibrosarcoma cells slightly increased in both samples over time. At 48 h after cell seeding, the fractions of apoptotic cells increased by 3.2 ± 0.6% in FLs1 cells and by 5.7 ± 1.3% in NLSdel cells (Fig. 3a). At 72 h after seeding, the fractions of apoptotic cells increased by 4.7 ± 2.3% and 6.7 ± 2.2%, respectively (Fig. 3b).

### Differential gene and protein expression in the presence and absence of nuclear syndecan-1

To provide new insight into the regulatory pathways governed by nuclear syndecan-1 we performed transcriptomic and proteomic screenings of the B6FS fibrosarcoma cells transfected with the three syndecan-1 constructs.

Nuclear translocation of syndecan-1 resulted in 20 differentially expressed genes compared to the deletion mutant unable to translocate to the nucleus. Of these, 2 genes were downregulated and 18 were upregulated (Table 1).

**Table 1:** Differentially expressed genes in cells with nuclear syndecan-1 (FLs1) versus cells with syndecan-1 lacking the NLS signal (NLSdel)

FLs1 vs NLSdel			
Symbol	Gene name	FC	q
EFCAB6	EF-hand calcium binding domain 6	2.24	0.013
CCKAR	cholecystokinin A receptor	1.97	0.025
EGR1	early growth response 1	1.85	0.003
CDCP1	CUB domain containing protein 1	1.84	0.00
ZNF676	zinc finger protein 676	1.84	0.031
NEK11	NIMA (never in mitosis gene a- related kinase 11	1.77	0.001
SLC16A4	solute carrier family 16, member 4 (monocarboxylic acid transporter 5)	1.75	0.013
DOCK8	dedicator of cytokinesis 8	1.72	0.00
LMBRD2	LMBR1 domain containing 2	1.68	0.037
SNORA56	small nucleolar RNA, H/ACA box 56	1.67	0.015
LPCAT2	lysophosphatidylcholine acyltransferase 2	1.6	0.017
LSM14B	LSM14B, SCD6 homolog B (*S. cerevisiae*)	1.58	0.047
HBG1	hemoglobin, gamma A	1.57	0.01
SNORA13	small nucleolar RNA, H/ACA box 13	1.57	0.026
SNORD116–6	small nucleolar RNA, C/D box 116–6	1.57	0.00
CCDC88C	coiled-coil domain containing 88C	1.56	0.03
EHF	ets homologous factor	1.52	0.006
PMFBP1	polyamine modulated factor 1 binding protein 1	1.51	0.015
PLA2G5	phospholipase A2, group V	−1.59	0.002
CYP3A7	cytochrome P450, family 3, subfamily A, polypeptide 7	−1.6	0.001

Differentially expressed genes identified by microarray analysis in fibrosarcoma cells with nuclear syndecan-1 (FLs1) versus syndecan-1 lacking the NLS signal (NLSdel). *FC* fold change, *q* false discovery rate

We successfully validated three significantly altered genes by RT-qPCR (Table 2a)**:** early growth response 1 (EGR1), never in mitosis gene a-related kinase 11 (NEK11), and dedicator of cytokinesis 8 (DOCK8). The first two proteins encoded by these genes are localized to the nucleus, whereas DOCK8 is mostly cytosolic.

**Table 2:** Differentially expressed genes from Affymetrix array, validated by qRT-PCR

Comparison	Gene product	Microarray (FC)	qPCR (FC, relative expression ± SD, *P*-value)
a. FLs1 vs NLSdel	EGR1 NEK11 DOCK8	1.85 1.77 1.72	1.39 ± 0.06 (*p* = 0.0004) 1.67 ± 0.41 (*p* = 0.04) 2.13 ± 0.49 (*p* = 0.08)
b. FLs1 vs V	COL1A2 PCDH18 ITGA8 PIP5K1B DACH1 AREG SERPINB4	0.58 0.54 0.48 0.47 0.41 0.40 0.36	0.51 ± 0,07 (*p* = 0.00001) 0.46 ± 0,04 (*p* = 0.00001) 0.50 ± 0.22 (*p* = 0.0019) 0.28 ± 0.18 (*p* = 0.0001) 0.29 ± 0.09 (*p* = 0.0001) 0.28 ± 0.25 (*p* = 0.0005) 0.21 ± 0.13 (*p* = 0.0005)
c. NLSdel vs V	COL19A1 FAP SERPINA3 SERPINB4 IL2RB	0.89 0.59 0.48 0.37 0.33	0.53 ± 0.37 (*p* = 0.02) 0.67 ± 0.07 (*p* = 0.00003) 0.59 ± 0.26 (*p* = 0.009) 0.66 ± 0.24 (*p* = 0.03) 0.55 ± 0.25 (*p* = 0.02)

Quantitative real-time PCR analysis of gene expression in B6FS cells overexpressing full-length syndecan-1 (FLs1), cells overexpressing NLS deleted syndecan-1 (NLSdel) and control cells (V). A subset of genes fulfilling the criteria of >1.5 fold up- or downregulation and a false discovery rate (q) ≤0.05 were further analyzed by qPCR. The table shows the significantly altered genes by both microarray and qPCR. *FC* fold change, *SD*-standard deviation. qPCR was performed three times, each in triplicates

The overexpression of FLs1 resulted in the modulation of 119 genes compared to control cells transfected with empty vector. Of these, 63 genes were downregulated. Following validation, we found that several matrix and membrane related proteins (e.g., AREG, COL1A2, PCDH18, SERPINB4) showed altered expression; in addition, several intracellular and nuclear factors were affected that had roles in signaling and cell growth, including DACH1, ITGA8, and PIP5K1B (Table 2b). Compared to control cells (V), the overexpression of NLSdel resulted in alterations in 42 genes, and all were downregulated. These genes encoded several secreted proteins, including COL19A1, FAP, IL2RB, SERPINA3, SERPINB4, and IL2RB (Table 2c).

With MS-based proteomics, we identified and quantified 8963 proteins across all samples. Changes in the proteome were modest, but the NLSdel cells showed more pronounced proteomic changes than the FLs1-transfected cells (both normalized to mock-transfected controls). At most, 0.5% of the detected proteins showed expression changes that exceeded 1.5-fold in individual samples (Additional file 1: Figure S4a). All except two replicates showed good Pearson correlations (*r* > 0.5) with their respective cluster groups (Additional file 4: Figure S4b and Additional file 5: Figure S5).

When the two non-clustering samples were excluded, the number of proteins regulated differentially between the FLs1 and the NLSdel groups increased from 21 to 122 at 1.5-fold changes and from none to 40 at 2-fold changes. Of the initial 21 proteins that were differentially regulated, 15 remained detectable after excluding the two non-clustering samples (Additional file 6: File S1). Based on these initial findings, we decided to perform subsequent analyses on a two-by-two sample basis.

### Upstream and downstream signaling events regulated by nuclear translocation of syndecan-1

**Fig. 4:**
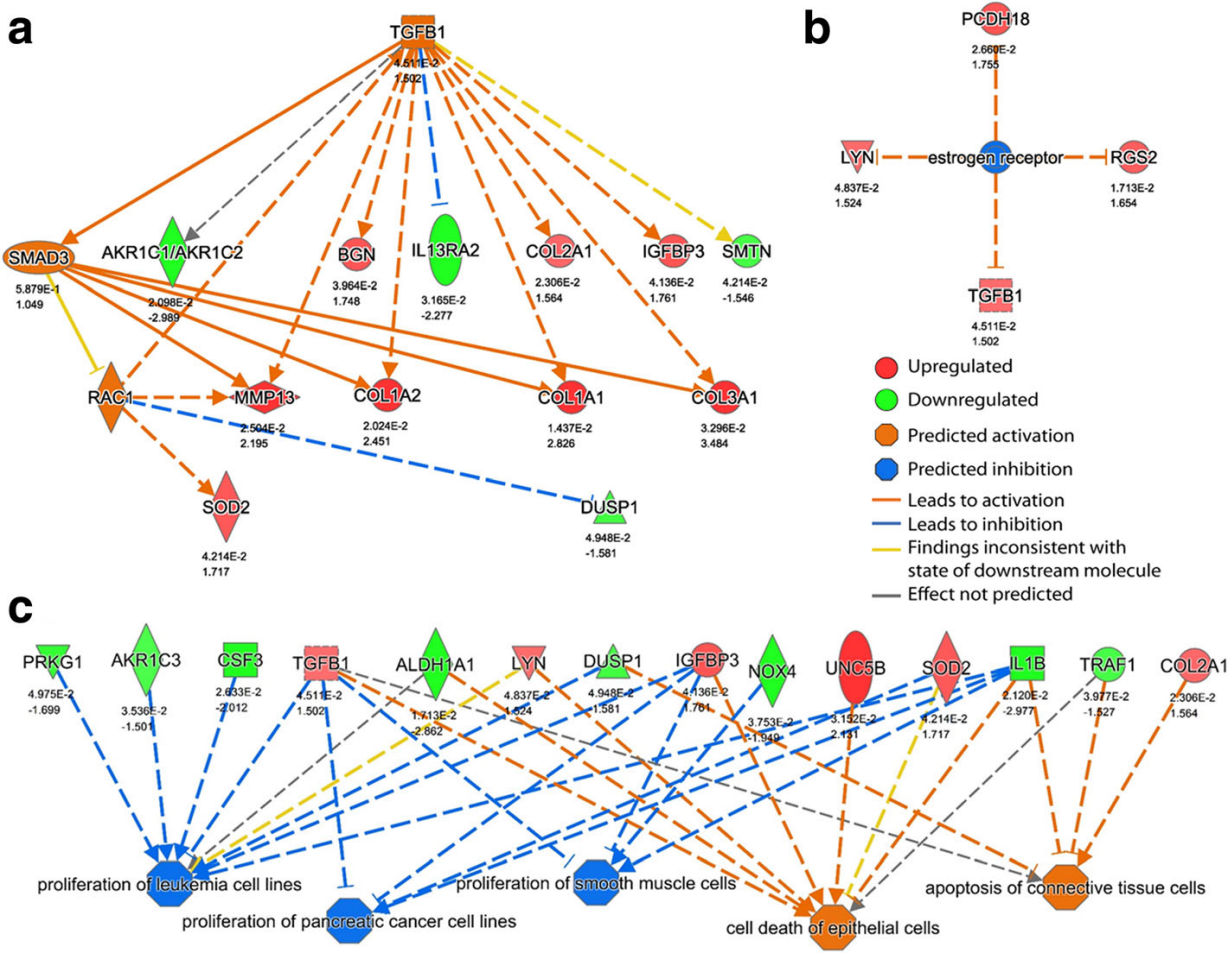
Upstream regulators and downstream biological effects predicted by Ingenuity Pathway Analysis on the proteome level. The “master regulators” of the analyzed dataset were predicted (blue and orange octagons), based on regulated proteins (green and red symbols indicate a fold change > |1.5| and a q-value <0.05) and literature data from the Ingenuity Knowledge Data Base. (**a**) TGF-β1, SMAD3, and RAC1 were the only upstream regulators predicted to be active, and (**b**) the estrogen receptor (group) was the only regulator predicted to be inhibited, when the full length syndecan-1 was expressed. (**c**) Regulated proteins were predicted to lead to a set of downstream biological effects, all pertaining to cell viability. All predicted regulators had a Z-score (activation score) > |1.9|, and a Fisher’s exact *p*-value < 0.05. Dashed lines are indirect effects, and the shape of the protein indicates the protein class (defined by IPA). Magnitude of regulations: TGF-β1 (Z-score = 2.62, *P*-value = 1.42e-06), SMAD3 (Z-score = 1.98, *P*-value = 1.38e-06), RAC1 (Z-score = 1.96, *P*-value = 2.15e-04), and the estrogen receptor (group) (Z-score = −2.0, *P*-value = 2.54e-02); proliferation of leukemia cell lines (Z-score = −2.08, *P*-value = 4.59e-06), proliferation of pancreatic cancer cell lines (Z-score = −1.96, *P*-value = 3.38e-03), proliferation of smooth muscle cells (Z-score = −1.95, *P*-value = 5.27e-03); death of epithelial cells (Z-score = 1.99, *P*-value = 5.96e-04), and apoptosis of connective tissue cells (Z-score = 1.91, *P*-value = 1.01e-03)

Because few transcripts were regulated, we have presented only proteomic results from the upstream and downstream regulatory analyses performed with the IPA, which compared FLs1 and NLSdel expression. Based on the pattern of differential protein expression, the IPA analyses indicated that TGF-β1, SMAD3, and RAC1 were activated. TGF-β1 was overexpressed in the FLs1 sample compared to NLSdel sample (1.5fold change, q < 0.05; Fig. 4a). The only regulator predicted to be inhibited by nuclear syndecan-1 was the estrogen receptor group (Fig. 4b). The proteomic data patterns predicted a consistent downstream biological effect, where cell death was activated and cell proliferation was inhibited (Fig. 4c).

**Fig. 5:**
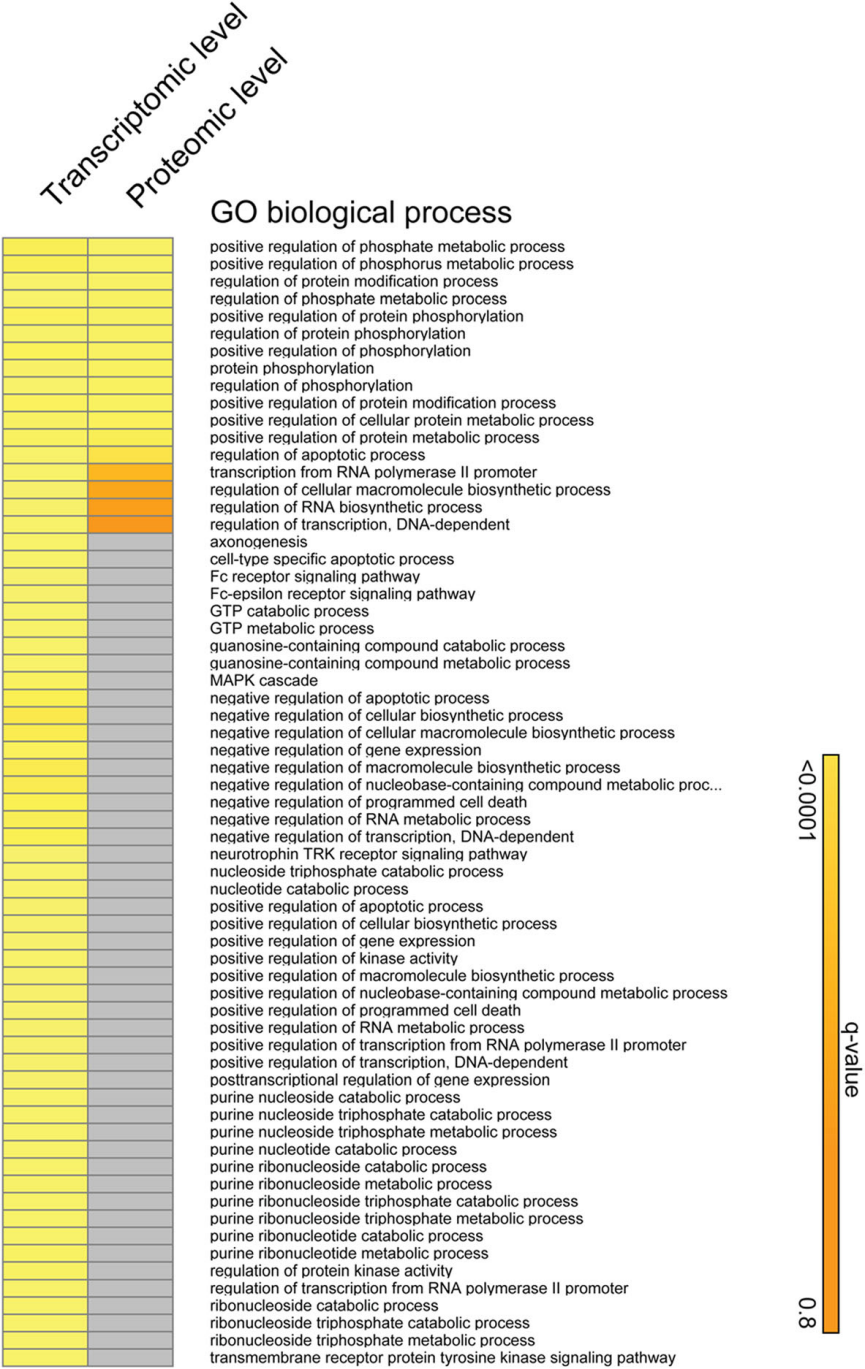
Network enrichment analysis highlights the overlap between transcriptomic and proteomic data. The most regulated transcripts or proteins (fold changes > |1.5| and q-values <0.05 between cells transfected with either syndecan-1 or syndecan-1 without a nuclear localization signal) were used separately to assess *GO biological processes* (Funcoup 3.0). The results were diagramed with the Gene-E program. Grey cells represent missing values

**Fig. 6:**
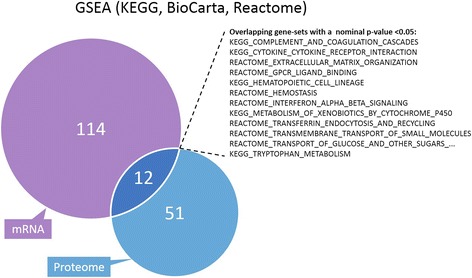
GSEA analyses show overlapping gene-sets significantly enriched with changes in the transcriptome (mRNA) and proteome. The pre-ranked GSEA analyses, with all genes ranked by their adjusted *p*-values (−log_10_ transformed) from two-sample moderated t-tests, between FLs1 and NLSdel. GSEA analyses were conducted separately for the transcriptome (mRNA) and proteome, with the KEGG, BioCarta, and Reactome databases

Although we found no overlap between the differentially regulated transcripts and proteins, our analysis of associated GO terms showed considerable overlap in biological processes. The networks generated by this method contained both the differentially expressed genes and their binding partners, and the lack of NLS was associated with several cellular functions. These data indicated a strong effect on protein modifications, particularly protein phosphorylation, transcription regulation, and apoptosis (Fig. 5). The GSEA analysis performed on the transcriptome of NLSdel versus Fls1 identified 114 pathways that were significantly enriched, following nuclear translocation of syndecan-1, and 51 pathways were identified by analyzing the proteome dataset. The overlap between the two analyses contained 12 pathways (Fig. 6), which depicted the common effects of syndecan-1 nuclear translocation on mRNA and protein levels. In the mRNA dataset, most of the significantly enriched pathways belonged to the categories of cell cycle regulation (13 pathways), DNA synthesis and transcription (10 pathways), and immune responses (9 pathways) (Table 3). In contrast, the top enriched pathways in the proteome dataset were related to cell adhesion and cell membrane transport; these functions were previously associated with syndecan-1. Interestingly, the proteome dataset also indicated the enrichment of pathways related to the immune system. Several pathways related to TGF-β were also enriched in the proteome dataset (Table 4 and Additional file 7: File S2).

**Table 3:** Categories of top enriched pathways in transcriptomic datasets according to Gene Set Enrichment Analysis (GSEA)

Cell Cycle	SIZE	p-val	rank
REACTOME_G1_S_TRANSITION	100	0.004	20
REACTOME_MITOTIC_G1_G1_S_PHASES	124	0.01	30
REACTOME_M_G1_TRANSITION	72	0.01	31
REACTOME_S_PHASE	100	0.011	37
REACTOME_CELL_CYCLE_CHECKPOINTS	105	0.015	43
REACTOME_FGFR_LIGAND_BINDING_AND_ACTIVATION	22	0.022	62
REACTOME_CELL_CYCLE	384	0.022	64
REACTOME_CYCLIN_E_ASSOCIATED_EVENTS_DURING_G1_S_TRANSITION_	62	0.027	78
REACTOME_G2_M_CHECKPOINTS	35	0.036	96
REACTOME_MITOTIC_M_M_G1_PHASES	162	0.04	109
REACTOME_CDK_MEDIATED_PHOSPHORYLATION_AND_REMOVAL_OF_CDC6	46	0.041	111
REACTOME_P53_DEPENDENT_G1_DNA_DAMAGE_RESPONSE	53	0.044	114
BIOCARTA_EIF_PATHWAY	16	0.046	117
DNA synthesis and transcription	SIZE	p-val	rank
REACTOME_RNA_POL_I_PROMOTER_OPENING	55	≤0.001	1
REACTOME_SYNTHESIS_OF_DNA	84	0.003	16
REACTOME_RNA_POL_I_TRANSCRIPTION	79	0.009	29
REACTOME_DNA_STRAND_ELONGATION	30	0.013	42
KEGG_DNA_REPLICATION	36	0.015	44
REACTOME_DNA_REPLICATION	182	0.023	67
REACTOME_RNA_POLI_RNA_POLIII_AND_MITOCH._TRANSCRIPTION	112	0.025	70
REACTOME_ACTIVATION_OF_THE_PRE_REPLICATIVE_COMPLEX	24	0.026	73
REACTOME_ASSEMBLY_OF_THE_PRE_REPLICATIVE_COMPLEX	57	0.026	75
REACTOME_TRANSCRIPTION	189	0.035	92
Immune System	SIZE	p-val	rank
REACTOME_INTERFERON_SIGNALING	151	0.017	47
BIOCARTA_CYTOKINE_PATHWAY	21	0.025	71
BIOCARTA_TH1TH2_PATHWAY	18	0.026	72
REACTOME_DOWNSTREAM_SIGNALING_EVENTS_OF_B-CELL_RECEPTOR_BCR	92	0.03	83
BIOCARTA_NKCELLS_PATHWAY	20	0.032	86
REACTOME_ANTIVIRAL_MECHANISM_BY_IFN_STIMULATED_GENES	64	0.034	90
REACTOME_CYTOKINE_SIGNALING_IN_IMMUNE_SYSTEM	260	0.037	99
BIOCARTA_DC_PATHWAY	22	0.044	115
REACTOME_SIGNALING_BY_THE_B_CELL_RECEPTOR_BCR	121	0.049	125

The GSEA analysis was performed on the transcriptome of NLSdel versus Fls1. The significantly enriched pathways belong to three categories: cell cycle regulation, DNA synthesis and transcription, and immune responses

**Table 4:** Categories of top enriched pathways identified by Gene Set Enrichment Analysis (GSEA) of proteomic dataset

Functions related to syndecan-1	SIZE	p-val	rank
REACTOME_COLLAGEN_FORMATION	28	≤0.001	1
REACTOME_NCAM1_INTERACTIONS	16	≤0.001	2
REACTOME_EXTRACELLULAR_MATRIX_ORGANIZATION	38	≤0.001	3
KEGG_ECM_RECEPTOR_INTERACTION	51	≤0.001	4
REACTOME_KERATAN_SULFATE_KERATIN_METABOLISM	16	0.002	10
REACTOME_CHONDROITIN_SULFATE_DERMATAN_ SULFATE_METABOLISM	29	0.001	16
REACTOME_INTEGRIN_CELL_SURFACE_INTERACTIONS	47	≤0.001	17
KEGG_CELL_ADHESION_MOLECULES_CAMS	47	≤0.001	
REACTOME_GLYCOSAMINOGLYCAN_METABOLISM	57	0.001	28
KEGG_FOCAL_ADHESION	137	≤0.001	34
REACTOME_TRANSMEMBRANE_TRANSPORT_OF_SMALL_MOLECULES	154	0.001	41
REACTOME_CELL_JUNCTION_ORGANIZATION	36	0.032	42
REACTOME_HEPARAN_SULFATE_HEPARIN_HS_GAG_METABOLISM	27	0.045	56
TGF β	SIZE	p-val	rank
REACTOME_DOWNREGULATION_OF_TGF_BETA_RECEPTOR_SIGNALING	17	0.014	26
REACTOME_TGF_BETA_RECEPTOR_SIGNALING_ACTIVATES_SMADS	19	0.018	27
KEGG_TGF_BETA_SIGNALING_PATHWAY	44	0.019	45
Immune System	SIZE	p-val	rank
KEGG_HEMATOPOIETIC_CELL_LINEAGE	25	0.001	12
REACTOME_INTERFERON_GAMMA_SIGNALING	32	0.001	15
REACTOME_IMMUNOREG_INTERACTIONS_BETWEEN_A_LYMPHOID_ AND_A_NON_LYMPHOID_CELL	16	0.005	18
BIOCARTA_IL1R_PATHWAY	24	0.001	23
REACTOME_IL1_SIGNALING	27	0.014	33
KEGG_NATURAL_KILLER_CELL_MEDIATED_CYTOTOXICITY	58	0.023	63

The GSEA analysis was performed on the proteomic dataset of NLSdel versus Fls1. Most of the top enriched pathways belong to categories already associated with syndecan-1. In addition, functions related to TGF β signaling and immune regulation were significantly enriched

### Sub-cellular localization of differentially regulated transcripts and proteins

**Fig. 7:**
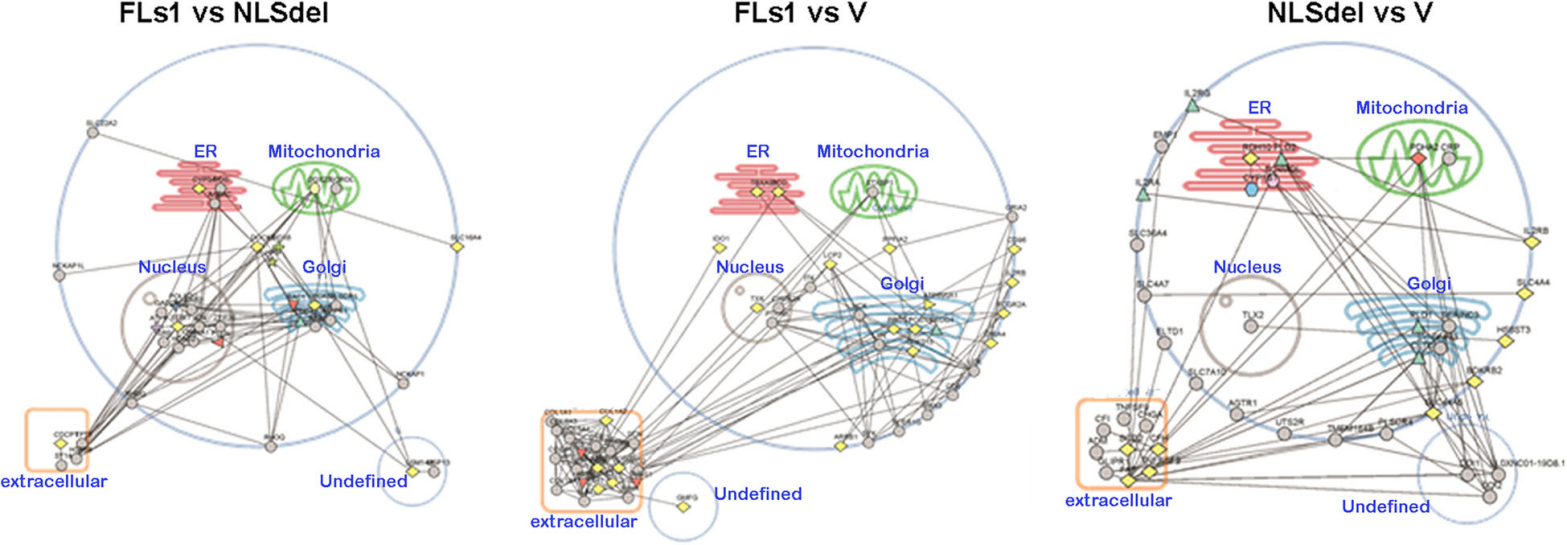
Subcellular localization of gene products regulated by the different constructs. Left: Comparison between the full-length syndecan-1 (FLs1) and the syndecan-1 lacking the nuclear localization signal (NLSdel) showed that most differentially expressed genes were localized to the nucleus. Middle: Comparison between FLs1 and the empty vector control (V) shows differentially expressed genes that code for membrane-bound proteins or proteins located to the extracellular matrix. Right: Comparison between the NLSdel and empty vector control (V) showed that most of the differentially expressed genes pertained to regulation of proteins in the cell membrane and extracellular matrix

Next, we studied the possible subcellular co-localization of the network components. When we compared the FLs1 and NLSdel transcriptome datasets, the proteins encoded by the most enriched genes (i.e., enriched GO cellular components) were nuclear. In contrast, when we compared either the FLs1 versus V or the NLSdel versus V datasets, the cytoplasm and membrane-related components and processes were enriched (Fig. 7).

### Transcription factors regulating cell growth are affected by nuclear translocation of syndecan-1

**Fig. 8:**
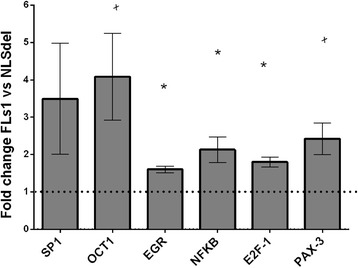
Cell growth-related transcription factors regulated by the nuclear translocation of syndecan-1. Results are shown from a TranSignal™ Protein-DNA array, performed with nuclear extracts from cells transfected with full-length syndecan-1 (FLs1) and syndecan-1 lacking the nuclear localization signal (NLSdel). Transcription factors expressed at levels that exceeded a 1.5-fold change were considered differentially expressed. The bars represent the average of at least two experiments; error bars represent the SEM. * p < 0.05, based on one-sample t-test against the theoretical value 1. ^x^ *p* < 0.1 indicates a trend observed in all three experiments (*p* = 0.056 for OCT1 and 0.08 for PAX3)

To elucidate the early events leading to these complex changes, we profiled the activity of cell-growth-related transcription factors that were affected by the nuclear translocation of syndecan-1. We extracted the nuclear proteins from both FLs1 and NLSdel transfected cells and hybridized them with a panel of consensus sequences that represented transcription factors with important roles in cell growth. The transcription factors with altered expression that were identified in repeated experiments are shown in Fig. 8.

The nuclear translocation of syndecan-1 significantly activated the nuclear factor kappa-light-chain-enhancer of activated B cells (NFκβ), E2F transcription factor 1 (E2F-1), and EGR. The activities of the POU domain, class 2, transcription factor 1 (OCT-1), paired box 3 (Pax-3), and Specificity Protein 1 (Sp1) were also altered in all three experiments, but the activation levels did not reach significance.

## Discussion

Syndecan-1 is critically involved in tumor cell proliferation and migration in a wide range of malignancies. The effects of syndecan-1 are tissue-dependent and largely vary in tumors of different origin [24, 25, 36–39]. The sub-cellular localization of syndecan-1 is important in this context. Syndecan-1 can be anchored to the cell membrane, translocated to the nucleus or shed and these locations have profound influences on its functions [40, 41]. Nikolova et al. found that the soluble syndecan-1 affects proliferation and invasiveness of breast cancer cells associated to a molecular signature including downregulation of TIMP-1, alteration in levels of uPAR, the Rho family of small guanosine triphosphatases and of integrins [41].

To test the functions and molecular pathways related to nuclear localization, we separately studied the functions of syndecan-1 in the nucleus and on the cell surface by employing a fibrosarcoma model, with preserved and impaired nuclear localization [25]. With this model system, we studied the functions regulated by the nuclear translocation of syndecan-1, with focus on cell growth. We combined transcriptomic and proteomic approaches to map the molecular mechanisms governing these functions on a global scale. The fact that most of the differentially expressed genes found in the omics screenings, were validated mainly by qPCR and only a few of them by a transcription factor array might constitute a limitation.

Previously, we have shown that syndecan-1 translocated to the nucleus in a regulated manner [7]. Here we demonstrate for the first time that the nuclear translocation of syndecan-1 has anti-proliferative effects; as cells with abolished nuclear localization proliferated at a significantly higher rate than those transfected with the full-length syndecan-1. Moreover, cells with nuclear syndecan-1 accumulated in the G0/G1 phase of the cell cycle at a higher extent compared to those with impaired nuclear localization. Ki-67 staining did not show significant differences among different constructs, all having a high proliferation rate in vitro.

There are evidences supporting both anti- and proapoptotic effects of syndecan-1 in different cell types. In myeloma syndecan-1 inhibited apoptosis [42] while its knock-down resulted in increase of apoptosis in endometrial cells [43], myeloma [44] and urothelial carcinoma cells [45]. Interestingly, in our experimental setting, the growth inhibitory effect of nuclear syndecan-1 compared to the cells with abolished nuclear translocation was accompanied by inhibition of spontaneous apoptosis, indicating that these two mechanisms might be interlinked. Similarly, Cortes et al. observed that overexpression of cell surface syndecan-1 in hepatocytes was associated with increased cell proliferation and apoptosis [46]. On the other hand, proliferation might be induced in the neighborhood of apoptotic cells as a compensatory mechanism, where, although apoptosis is initiated, the effector caspases are inhibited, and thus, the living cells constantly emit mitogenic signals, which stimulate the surrounding cells to proliferate [47, 48]*.*

The subcellular localization of syndecan-1 elicited a plethora of molecular changes which were categorized and analyzed by means of extensive bioinformatics. Network analyses pointed predominantly toward altered genes and pathways related to the nuclear compartment. In accordance with our earlier data [22, 49, 50], we found that syndecan-1 overexpression altered TGF-β-related signaling pathways and cell cycle regulation. Moreover, the TGF-β pathway was predicted by bioinformatics as master regulator associated with the nuclear translocation of syndecan-1 in this setting. In mesothelioma cells TGF-β inhibited the nuclear translocation of syndecan-1, and this inhibition hampered the proliferation of the cells [51].

We identified three genes that were significantly enhanced by the nuclear translocation of syndecan-1: EGR-1, NEK11, and DOCK8, suggesting that these genes are responsive to the nuclear translocation of syndecan-1. However, it remains to be determined whether they are direct targets or mediators of syndecan-1 effect in the nucleus.

EGR-1 is a transcription factor activated by a wide variety of extracellular stimuli and apoptotic signals. NEK11 is a DNA damage-response protein. Both proteins are localized in the nucleus and play multiple roles in the cell cycle. NEK11 kinase activity directly phosphorylates CDC25A; thus, it is required for DNA damage-induced G2/M arrest [52]. NEK11 in turn is dependent on the cell cycle; its highest expression occurs in the G2/M phase [53], but its activation through an association with Nek2A is enhanced in G1/S-arrested cells [54]. Similar to NEK11, EGR-1 is important in cell cycle progression: it regulates the G0/G1 transition [55], and it activates cyclin D2 [56]. Thus, it plays a role in the G1/S transition, and increases entry into the S/G2-phase. NEK11 is primarily associated with DNA replication and damage, stress-responses, and drug resistance [53, 57]; it is activated by DNA-damaging agents and DNA replication inhibitors [52, 53]. In our experimental setting, nuclear syndecan-1 activated NEK11, and thus, inhibited cell proliferation by causing cell cycle arrest.

In some tumor types, EGR-1 promotes growth and induces resistance to apoptosis [56]. In other tumor types, it can promote apoptosis [58] and significantly suppress tumor growth [59]. In HT1080 fibrosarcoma cells, tumor suppression is associated with inhibition of p53-dependent apoptosis [60]. EGR-1 regulates multiple tumor suppressors, in addition to p53, including TGF-β and PTEN [61]. Moreover, there is a complex relationship between TGF-β and EGR-1. In kidney [62] and colon cancer cells, EGR-1 induced TGF-β1 to suppress growth and tumorigenicity. In HT1080 fibrosarcoma cells, TGF-β induction was associated with increased adhesion [63]. Interestingly, in non-small lung cancer, EGR-1 counteracted the TGF-β-induced epithelial-to-mesenchymal transition [64]. In turn, EGR-1 was identified as a TGF-β target [65, 66]. In our study, nuclear syndecan-1 induced EGR-1 expression, which was associated with activating the TGF-β pathway and a slight inhibition of apoptosis. These features could be interconnected. The induction of EGR-1 expression by nuclear syndecan-1 was a very consistent result throughout our experiments. Our assessment of the activation of cell growth-related transcription factors showed that the magnitude of EGR-1 activation was in concordance to its upregulation at the RNA level, based on the Affymetrix array and the qPCR results.

DOCK8 is a member of a guanine nucleotide exchange factors family, involved in regulating cell morphology and intracellular signaling. It interacts with the Rho GTPase Cdc42 [67], and acts as a guanine nucleotide exchange factor [68]; thus, its deficiency might lead to impaired tumor immune surveillance. DOCK8 also participates in regulating tumor cell invasion [69] and metastatic processes [70]. Its expression was reduced in lung cancer [71] and altered in gliomas [72].

Our data also point toward several other transcription factors that were differentially regulated by nuclear syndecan-1. Some of these factors could directly activate the basic transcription machinery, like SP1 and E2F-1 [73]. SP1 induces apoptosis and inhibits cell cycle progression. E2F-1 is required for entry into the S1 phase [74]. Syndecan-1 is regulated by SP1, as the promoter of the syndecan-1 gene contains a SP1 binding site [75]. EGR-1 also interacts with other transcription factors and can compete with SP1, activate NFκβ and AP-1 [76], and in turn, it is activated by NFκβ and by E2F-1 [77].

Among these transcription factors, only NFκβ was previously associated with syndecan-1. One study showed reduced cellular NFκβ levels, when syndecan-1 was silenced [78]. NFκβ is considered a positive mediator of cell growth [79]. Its growth-promoting effects are typically associated with the inhibition of apoptosis. However, it was also demonstrated that environmental signals determine whether NFκβ induction leads to apoptosis or survival [80]. The dependency upon environmental factors is valid for most transcription factors as they may play context-dependent and dual roles in mediating cell growth and apoptosis. For example, E2F-1 knockout mice developed tumors [81], despite the fact that E2F-1 is crucial for progression through the S-phase. This result could be explained by the fact that E2F-1 is also important in apoptosis [82]. NFκβ stimulates proliferation in some environments [83], but leads to apoptosis in others [82]. OCT-1 could mediate growth arrest in some tissue types [84]; in other settings, low OCT-1 levels activated IFN-g and had pro-proliferative effects, but high OCT-1 levels had pro-apoptotic effects [85].

**Fig. 9:**
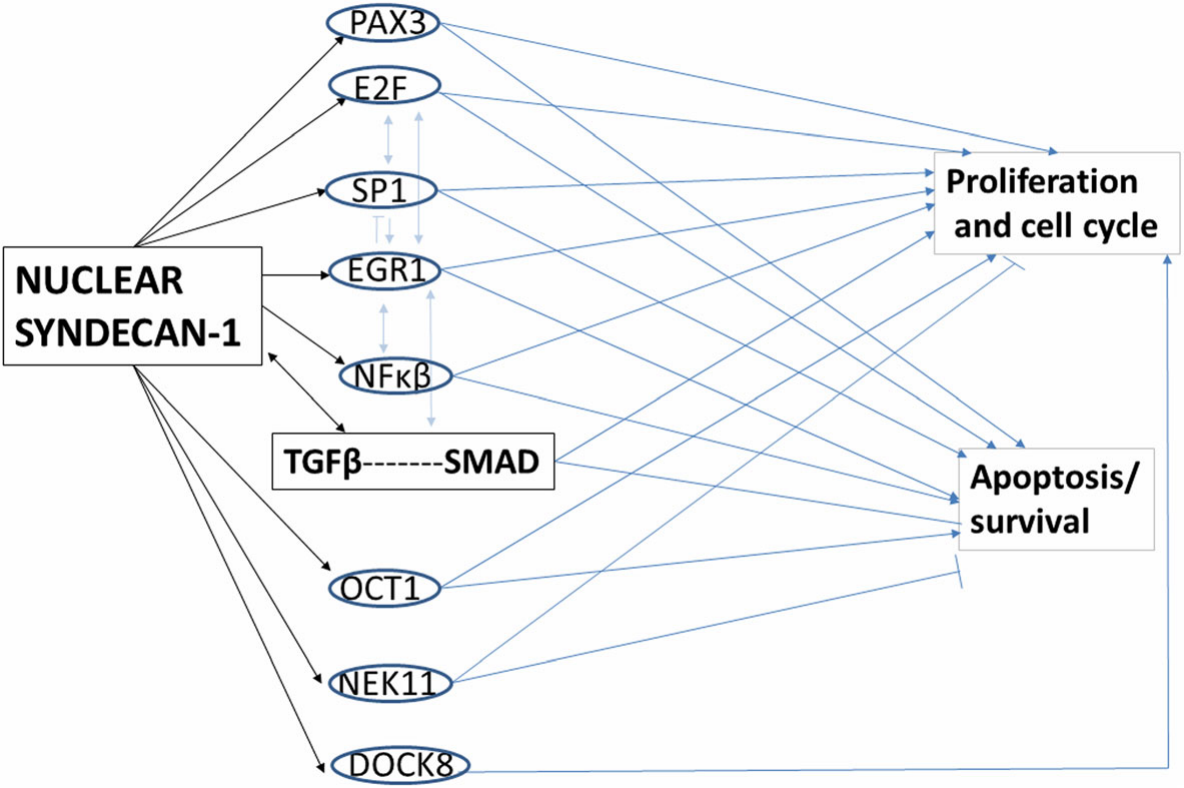
Regulatory network elicited by nuclear translocation of syndecan-1 leading to inhibition of proliferation and survival. Data are based on differentially expressed genes obtained by microarray and transcription factor array on fibrosarcoma cells with preserved and impaired nuclear translocation of syndecan-1. Nuclear syndecan-1 activates several transcription factors (ovals) and induces immediate early genes. Transcription factors activate (arrows) or inhibit (bars) their targets, and in addition, they trigger the TGF-β pathway (rectangle). In turn, TGF-β can also induce EGR-1 and other transcription factors, which can provide a feedback loop. The arrows in blue represent the current knowledge about the role of target genes in governing different processes. The functional outcome of nuclear syndecan-1 signaling is the measured inhibition of proliferation and cell survival

The effect of nuclear syndecan-1 seem to be consistent with these observations: by co-activating several transcription factors, nuclear syndecan-1 initiates a series of molecular events, which ultimately lead to the inhibition of both proliferation and apoptosis (Fig. 9). Our findings are consistent with recently described processes, where first, transcription factors activate proliferation-related genes at relatively low levels; then later, as the transcription factors accumulate, apoptosis-related genes are activated. Other factors, like histone-modifying genes or microRNAs could also affect the timing of this process. This theory is based on observations that the same transcription factors are involved in both proliferation and apoptosis [82, 86].

Although the deletion of the syndecan-1 nuclear localization signal caused only modest net changes in the transcriptome and proteome, the effects were considerable, bearing in mind that only a pentameric amino acid sequence was removed. All the elicited changes clustered in distinct patterns, which indicated the functional importance of nuclear syndecan-1. The overlap between the differentially regulated transcripts and proteins were limited, which may be related to the kinetics of nuclear translocation and transcription. When the cells were trypsinized and seeded, they lost all syndecan-1 proteins, in both the cell membrane and nucleus; however, at 48 h after seeding, this proteoglycan could be detected in the nucleus [7]. At this time point, some syndecan-1-regulated transcripts may not have completed translation. However, when we evaluated the effects of the regulated transcripts and proteins in GO terms, we found an overlap in the biological processes affected by syndecan-1. These processes were dominated by phosphorylation and other post-translation protein modifications, which indicated alterations in intracellular signaling. Additionally, we found overlapping enrichments in several KEGG and Reactome pathways related to extracellular matrix organization, transmembrane transport, and endocytosis. These pathways played roles in functions previously associated with syndecan-1. Thus, the effects on the proteome were related to several known functions of syndecan-1. In contrast, the effects on transcription were linked to gene expression and cell cycle control.

With the IPA, we dissected the proteomic changes in more detail by linking significantly regulated proteins to certain *regulators*; then, by inferring literature-based directionality, we could determine whether these regulators were predicted to be activated or inhibited. We found a clear pattern of significantly regulated proteins that were related to an active TGF-β1/SMAD3/RAC1 axis. The connection between syndecan-1 and TGF-β1 was reported previously [49, 87, 88], and this pathway represented the only regulators significantly activated in our dataset. Importantly, the outcomes of the TGF- β-mediated signaling events were fine-tuned and highly dependent on the spatial distribution and the sub-cellular localization of various members of the signaling cascade. Independent studies have confirmed the inhibitory role of HS on TGF-β1 signaling; it facilitated lipid raft/caveolae-mediated endocytosis and rapid degradation [89].

## Conclusion

We showed that nuclear syndecan-1 inhibited proliferation and cell cycle progression in fibrosarcoma cells. The global characterization of the transcriptome and proteome related to nuclear syndecan-1 indicated that these effects were delicately regulated by multiple actors in related signaling pathways, where TGF-β1 seemed to play a central role. The nuclear ligands of syndecan-1 and the subsequent signaling pathways should be further elucidated to clarify our understanding of the importance of this HSPG in the nucleus. Our study results suggest that EGR1, NEK11, and several other transcription factors such as NFκβ and E2F-1 are syndecan-1 targets in the nucleus.

## Methods

### Cell characteristics and culture conditions

**Fig. 10:**
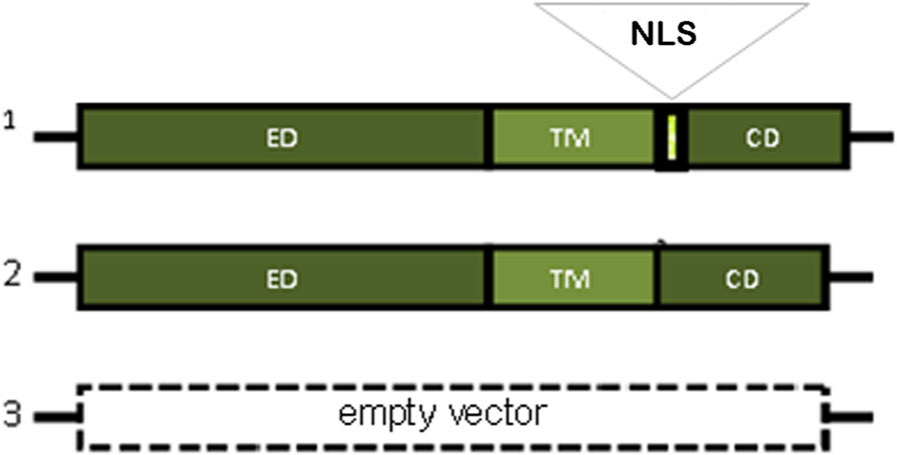
Plasmid constructs used for transfection. (**1)** Full length syndecan-1 (FLs1); (**2**) Syndecan-1 that lacked the nuclear localization signal (NLSdel); and (**3**) Empty vector control (V). ED = ectodomain, TM = transmembrane domain, CD = cytoplasmic domain

We used subtypes of a human fibrosarcoma cell line (B6FS) [90] that had low endogenous syndecan-1 levels, transfected with three different constructs: 1.) a plasmid carrying the full-length syndecan-1 gene (FLs1); 2.) the same plasmid carrying syndecan-1, but lacking the RMKKK nuclear localization signal (NLSdel), and 3.) the empty vector (V) as a control (Fig. 10). For detailed description of these plasmids and cell transfection see [25]. The stably transfected cells were cultured under selective pressure with Geneticin (G418, Roche Diagnostics GmbH, Mannheim, Germany). Previously, we showed that after transfection of FLs1, syndecan-1 was detected in the nucleus, whereas the nuclear translocation of syndecan-1 was hampered in NLSdel [25]. We cultured these cells in RPMI 1640-GlutamaxTM-I medium (72,400, Gibco) supplemented with 10% fetal bovine serum (FBS), under standard incubation conditions, in humidified 5% (*v*/v) CO2 at 37 °C.

We carefully controlled the experimental conditions to obtain similar levels of syndecan-1 expression throughout the experiments. We regularly verified the syndecan-1 levels by fluorescence activated cell sorting (FACS) and western blotting prior to RNA extractions and mass spectrometry. This verification ensured that the differences detected were related to the presence or lack of nuclear syndecan-1 and not to differences in syndecan-1 expression levels.

### Fluorescence activated cell sorting (FACS)

For FACS analyses, cells were detached with enzyme-free Cell Dissociation Buffer (Gibco, 13,151–014) for 15 min, and when necessary, cells were scraped from the plate. Cells were collected, counted, and fixed in 2% buffered formaldehyde. We then incubated the cells with antibodies against the ectodomain of syndecan-1 (MCA658) for 15 min at 4 °C. After washing, cells were stained with Alexa 488-conjugated goat anti-mouse secondary antibody (Molecular Probes, A-11001) for 15 min at room temperature (RT), in the dark. Subsequent experiments were performed when the syndecan-1 levels in both FLs1 and NLSdel cells ranged between 1.5- and 2-fold above the levels in control cells.

### Western blotting

For western blotting sub-confluent cells were dissociated with 0.5% Trypsin-EDTA and washed twice with PBS. Lysis was achieved by incubation for 15 min in buffer containing: 50 mM Tris-HCl, pH 8.0, with 150 mM sodium chloride, 1.0% Igepal CA-630 (NP-40), 0.5% sodium deoxycholate, 0.1% sodium dodecyl sulfate and protease inhibitor (Thermo scientific). Lysed cells were spun at 16000 x g for 5 min at 4 °C, and the supernatant was collected and mixed with 2X Laemmli loading buffer with 2-mercaptoethanol. Samples were separated by SDS-polyacrylamide gel electrophoresis. Transfer was performed to a PVDF membrane using the trans-blot turbo transfer system (Bio-Rad). The membrane was blocked for 1 h in 0.5% milk and incubated overnight at 4 °C with primary antibodies for Syndecan-1 (C-20) (Santa Cruz Biotechnology cat.nr. SC-7099) diluted 1:200, and monoclonal Anti-Actin (Clone AC-40) (Sigma-Aldrich, cat.nr. A4700) diluted 1:500. Following washes the membrane was incubated with secondary antibodies (Rabbit Anti-Goat IgG, F (ab’)_2_ Fragment Specific, Peroxidase Conjugated (Thermo scientific) and ECL™ Anti-mouse IgG, Horseradish peroxidase linked F(ab’)_2_ fragment (from sheep) (GE Healthcare) at 1:5000 dilution for 1 h at room temperature. For chemiluminescent detection, chemiluminescent HRP Substrate (Advansta, cat.nr. K-12043-D10) was added and the membrane was incubated for 1 min. The Odyssey Imaging System (LI-COR) was used to develop the membrane and the relative expression of syndecan-1 was normalized to actin as loading control using the ImageJ software.

### Immunocytochemical staining and subcellular localization of the newly synthesized syndecan-1

The subcellular localization and the level of syndecan-1 was further verified using immunocytochemical analysis and subsequent fluorescent microscopy, as described previously 25]. Cells were seeded on to POLYSINE coated microscopy slides (Menzel-Gläser, Braunschweig, Germany). After 48 h cells were fixed in 3% paraformaldehyde followed by permeabilization with 0.1% Triton X-100 (Sigma, Steinheim, Germany); non-specific binding was blocked with 3% goat serum (Dako A/S, Glostrup, Denmark) for 30 min. Mouse anti Human CD138 monoclonal antibody (MCA-681) Serotec LTD, Kidlington, Oxford, England) and mouse IgG1 (Dako A/S, Glostrup, Denmark)as negative control was used to stain syndecan-1, followed by incubation with Alexa 488 goat anti-mouse F(ab′)2 fragment of IgG (H + L), (Molecular Probes, Leiden, The Netherlands, A11017). Samples were then counterstained with 1 mg/L bisbenzimide H33342 (Fluka, Steinheim, Germany). Detailed visualization was performed using Nikon microphot-FXA EPI-FL3 fluorescence microscope.

### Cell proliferation assay

Different densities of FLs1 and NLSdel cells (2000, 3000, or 4000 cells/well) were seeded on 96-well plates. Cell proliferation was measured with the Cell Proliferation Reagent, WST-1 (Roche Diagnostics Scandinavia AB, Bromma, Sweden) at different time points, according to the manufacturer’s instructions. Briefly, cells were incubated with 1/10 (*v*/v) WST1 reagent for 2 h at 37 °C. Samples were analyzed with a Spectramax spectrophotometer at 450 nm with background subtraction at 630 nm. Three independent experiments were performed, each in triplicate. The paired Student’s t test was applied to determine statistical significance, with GraphPad Prism software. Doubling time was calculated from the logarithmic phase of the growth curve [91].

### Immunocytochemical detection of Ki-67

As an additional measure of proliferation, we used immunocytochemistry to label proliferating cells with the proliferation marker Ki-67. For this purpose, cytospin preparations of cells transfected with the three different constructs were done on SuperFrost Plus glass slides (Thermo Fisher Scientific Inc., Waltham, MA, USA). Cells were fixed in H2O with 25% ethanol, 25% methanol and 3% polyethylene glycol (PEG). Prior to staining, PEG was extracted by decreasing concentrations of ethanol in H2O. For epitope retrieval slides were incubated at 100 °C for 5 min in a citrate buffer pH 6.0 (Bond Epitope Retrieval Solution 1, Leica Microsystems GmbH). Endogenous peroxidase activity was abolished with 3% hydrogen peroxide in H2O. Slides were then incubated for 30 min with primary antibody (Dako M7240) diluted 1:200 in BOND Primary Antibody Diluent (Leica Microsystems GmbH). Secondary IgG was added and incubated for 15 min and detected with the Bond Polymer Refine Detection kit (Leica Microsystems GmbH); as described in the manufacturer’s protocol. Following 15 min incubation with a poly-HRP, bound antibodies were visualized by Diaminobenzidine. Cell nuclei were counterstain with hematoxylin. The immunostaining was performed in a Leica BOND-III automated IHC with relevant controls.

For each cell line, random microscopic fields have been photo documented and evaluated. For each construct, at least 200 cells were counted. The presence or absence of nuclear reactivity to Ki67 was recorded and the percentage of Ki-67 positive cells was related to the total number of cells.

### Cell cycle analysis

FLs1and NLSdel cells were grown for 48 h; then, they were harvested, fixed in 1 mL of 70% cold ethanol, and incubated at 4 °C overnight. Cells were washed in PBS, resuspended in 500 μL staining solution containing 50 μg/mL propidium iodide (Sigma-Aldrich, MO, USA) and 100 μg/mL RNAse A (Sigma-Aldrich, MO, USA), and incubated for 30 min at 37 °C. The cell cycle distribution was measured for 10,000 cells in each sample with a FACSCalibur cytometer (Becton Dickinson, CA, USA). Results were analyzed with ModFit LT software (Verity Software House, ME, USA). Statistical significance was tested with the paired t test in GraphPad software.

### Measurement of spontaneous apoptosis

We detected apoptosis by performing FACS analysis with the FITC Annexin V Apoptosis detection kit (BD Pharmingen), according to the manufacturer’s instructions. Briefly, cells were trypsinized, washed with PBS, and resuspended in Binding Buffer with Annexin V-FITC and propidium iodide. Then, cells were incubated for 15 min in the dark, followed by FACS analysis. The apoptosis was measured at 48 and 72 h after cell seeding. Three independent experiments were performed for both time points. Statistical significance was assessed with the paired t test, in GraphPad software.

### Transcriptomic and proteomic data generation

#### RNA isolation

At 48 h after seeding, we isolated total RNA from fibrosarcoma cells transfected with FLs1, NLSdel, or control V with the High Pure RNA Isolation Kit (Roche Diagnostics GmbH Mannheim, Germany), in accordance to the manufacturer’s protocol. Three biological replicates were used for each construct. The yield and purity of the RNA were determined by measuring the UV absorbance at 260 and 280 nm with a NanoDrop spectrophotometer (NanoDrop Technologies Inc.).

#### Affymetrix gene expression array

To disclose the molecular mechanisms underlying syndecan-1 nuclear translocation, we performed microarray analysis on cells that overexpressed FLs1 and NLSdel at similar levels. RNAs isolated from the cells were subjected to microarray analysis with the GeneChip Human Gene 1.1 ST Array (Affymetrix Inc., Santa Clara, CA, USA), which covered the whole transcript. Target synthesis and hybridization was performed in the Affymetrix core facility (Novum, Karolinska Institutet, Stockholm, Sweden). The raw data has been deposited in the MIAME compliant database Gene Expression Omnibus (accession number GSE81504). Image analysis and data pre-processing was performed with the Affymetrix Gene Chip Command Console. For data processing, we performed background correction with the PM-GCBG method (subtracting the GC-content specific background); data normalization with the Global Median method; and raw intensity value summarizations with PLIER (Probe Logarithmic Intensity Error). For each sample, the analysis generated a signal that represented the relative measure of transcript abundance. Individual signals that exceeded a value of 10 were considered for further analysis.

#### Preparation of cells for mass spectrometry-based proteomics

Cells were grown in 75 cm^2^ culture dishes for 48 h in triplicate. Then, cells were lysed with 4% SDS, 25 mM HEPES, and 1 mM DTT, on ice. Cell lysates were heated to 95 °C for 5 min, followed by 1 min sonication, and 15 min centrifugation at 14,000 g. Proteins were reduced, alkylated, and digested to peptides according to an adapted FASP protocol [92]. Individual samples were labeled with TMT-10plex isobaric labels (Thermo Fischer Scientific, San Jose, CA, USA), according to the manufacturer’s instructions. Briefly, 80 μg of peptides from each sample was combined with a designated TMT reagent, and labeling was performed at room temperature for 3 h. Labeling controls were preformed to guarantee >99% labeling of primary amines. Then, samples were combined (i.e., a total of 800 μg) and cleaned on a SCX column (Phenomenex, Torrance, CA, USA).

#### High resolution isoelectric focusing

We used isoelectric focusing to fractionate our TMT-10plexes, and thereby reduce the complexity of the proteome. Specifically, we applied the recently developed, high resolution, isoelectric focusing method (HiRIEF) [93]), with an immobilized pH gradient of 3.7 to 4.9 (kindly provided by GE healthcare, Uppsala, Sweden). The TMT pooled sample (390 μg) was applied to the HiRIEF strip and run on an Ettan IPGphor (GE Healthcare) until at least 100 kVh had been reached (around 24 h). The fractionated sample was extracted from the gel strip in an automated manner, to yield 72 individual fractions. These fractions were then injected separately on a Q Exactive mass spectrometer (see section 2.6.5). This procedure was previously described in more detail [93].

#### nanoLC-MS/MS analysis

Peptides were separated with an online 3000 RSLCnano system. Samples were trapped on an Acclaim PepMap nanotrap column (C18, 3 μm, 100 Å, 75 μm × 20 mm), and separated on an Acclaim PepMap RSLC column (C18, 2 μm, 100 Å, 75 μm × 50 cm; Thermo scientific). Next, HiRIEF-fractionated peptides were separated on a gradient of A (5% DMSO, 0.1% Formic acid; FA) combined with B (90% Acetonitrile; ACN, 5% DMSO, 0.1% FA), where B ranged from 3% to 37%. Samples were run for 50 min at a flowrate of 0.25 μL/min. The Q Exactive instrument (Thermo Fischer Scientific, San Jose, CA, USA) was operated in a data-dependent manner, where the top 5 precursors were selected for HCD fragmentation and MS/MS. The survey scan was performed at 70,000 resolution over a range of 300–1600 *m/z,* with a maximum injection time of 100 ms and target of 1 × 10^6^ ions. HCD fragmentation spectra were generated with a maximum ion injection time of 150 ms and an AGC of 1 × 10^5^. Then, fragmentation was performed at 30% normalized collision energy, with 35,000 resolution. Precursors were isolated with a width of 2 *m/z* and placed on the exclusion list for 70 s. For 4-h gradients, we used a top 10 method, with a survey scan over the range of 400–1600 *m/z* and a maximum injection of 140 ms. Single and unassigned charge states were rejected from precursor selection.

### Data analysis and bioinformatics

#### Affymetrix data analysis

We performed a differential gene expression analysis, based on Affymetrix data, with the OCplus package provided in R software *(* *http://www.R-project.org/* *)*. [94] We conducted three pairwise comparisons, including FLs1 versus NLSdel, FLs1 versus V, and NLSdel versus V. We compared signals between samples with paired t-tests. The *p*-values were converted to false discovery rates (q-values) with a multiple-testing correction. A threshold of q ≤ 0.05 was applied, and differentially expressed genes were ranked by the fold-change (i. e., the ratio of expression values between a sample and a control). Thus, a syndecan-1 modulated sample was compared to its corresponding control. A transcript was considered significantly up- or down-regulated, when the fold change exceeded |1.5|. Probeset IDs were converted to HUGO gene symbols to denote the genes. We performed network enrichment analysis with Funcoup 3.0 network of functional coupling (http://funcoup.sbc.su.se) [95], in two different ways. In the first approach, we applied the functional analysis on the previously established, differentially expressed genes, for each pair of data. This method was suited to disclosing the possible involvement of differentially expressed genes in various cellular functions and to map their distribution to different cellular compartments. In the second approach, in addition to a differential analysis of the fold-change, we performed a global network analysis of functional coupling to reveal the involvement of genes with specific biological functions, which were apparent when syndecan-1 was overexpressed with or without the NLS. This approach allowed investigation of functional relationships between differentially expressed genes, particularly when summarizing small changes in many related genes. It also highlighted differentially expressed genes that might be direct binding partners of syndecan-1, based on currently available data from the literature available in the curated resources, Gene Ontology (GO), Reactome, and KEGG.

#### Peptide identification, protein identification, and data analysis

We used the Proteome discoverer 1.4 with Sequest HT and percolator search algorithms to construct the proteome. The precursor mass tolerance was set to 10 ppm, and to 0.02 Da for fragments. We set oxidized methionine as a dynamic modification, and we set carbamidomethylation of cysteines, TMT10 on the N-terminus, and lysines as fixed modifications. Spectra were matched to the Uniprot human database (downloaded 20,140,203), limited to a positive false-discovery rate (FDR) of 1%. The FDR was determined by searching against a decoy database of similar size with reversed sequences. All TMT10 quantifications were median-centered for each sample. FLs1 and NLSdel samples were normalized to the empty control V. A moderated t-test was performed to determine the number of proteins that were significantly changed between FLs1 and NLSdel samples. *P*-values were adjusted with the Benjamini-Hochberg correction (q-values). The moderated t-test was performed in the R software environment (version 3.1.2). As a quality control, samples were clustered (one minus the Pearson coefficient) with the Gene-E software platform (http://software.broadinstitute.org/morpheus/) [96]. The vast majority of the proteome remained unperturbed. This feature made clustering of samples on the global scale very susceptible to background fluctuations (technical or biological), and two of the replicates did not cluster together with their respective groups. Therefore, we decided to exclude these two samples from the final analyses and proceeded with the remaining four samples for subsequent analyses.

#### Bioinformatic analyses of the proteome

Proteins with fold-changes that exceeded |1.5| and with adjusted *p*-values (two-sample moderated t-test) < 0.05 were considered for analysis with Ingenuity Pathway Analysis (IPA, version 23,814,503; QIAGEN). Only findings with experimental observations in human cell lines or tissues were considered. Upstream and downstream *(disease and function)* analyses were performed with data from the IPA Knowledge Data-Base, which predicted the activation or inhibition of regulators or downstream biological effects [97]. These predictions were reported, and considered significant, when they had a Z-score > 1.9 for activation and <1.9 for inhibition. A Fisher’s exact p-value ≤0.05 was taken to indicate a significant overlap with upstream regulation or downstream biological effects. The upstream and downstream effects were discerned from the pattern of identified proteins, and the degree of consistency between the observed levels and those reported in the published scientific literature. Furthermore, we analyzed differentially regulated transcripts and proteins with Funcoup 3.0 to assess the overlap between findings in transcriptome and proteome spaces, based on the GO terms. These analyses were based on genes and proteins that were differentially expressed between FLs1 and NLSdel samples, with a fold-change that exceeded |1.5| and a q-value <0.05. Additionally, we performed a gene set enrichment analysis (GSEA; http://software.broadinstitute.org/gsea/index.jsp) [98] with a pre-ranked test, where all gene names were ranked by their adjusted *p*-values (−log_10_ transformed) from two-sample, moderated t-tests, between FLs1 and NLSdel. GSEA analyses were conducted separately for the transcriptome and proteome, with the KEGG, BioCarta, and Reactome databases.

### Validation and functional assays

#### RT-qPCR

We validated the Affymetrix results with real-time quantitative polymerase chain reaction (RT-qPCR) assays. cDNA synthesis was performed by reverse transcribing 2 mg RNA with a First-Strand cDNA Synthesis Kit (Amersham Pharmacia Biotech., Little Chalfont, Buckinghamshire, England). We used the same RNAs that were used for the Affymetrix analysis. We performed RT-qPCR with the Platinum SybrGreen qPCR SuperMix-UDG kit (Invitrogen) and DNA-polymerase, with a set of sense/antisense primers (CyberGene AB, Sweden).

The primers were designed based on gene sequences from GeneBank (NCBI), with the exception of GAPDH [99] and syndecan-1 [100]. The primer sequences are shown in Table 5. All PCR reactions were performed with an iCycler machine (CFX96TM Real Time PCR Detection System, BioRAD Hercules, CA, USA), in triplicate, with a total volume of 10 μL/well, and a primer concentration of 200 nM. We performed the analyses with Bio-Rad CFX Manager Software 2.0 (BioRad Laboratories 2008). Data were analyzed with the 2^-ΔΔCt^ method. Each target was normalized to GAPDH, as the reference gene, and the fold-change in expression was measured for each target with respect to the corresponding controls. The data are expressed as the mean of at least three independent experiments.

**Table 5:** Primer sequences used for RT-PCR validation

GENE	PRIMERS (5’TO 3’ORIENTATION): FORWARD/REVERSE
GAPDH	ACATCATCCCTGCCTCTACTGG/ AGTGGGTGTCGCTGTTGAAGTC [99]
SDC1	TCTGACAACTTCTCCGGCTC/CCACTTCTGGCAGGACTACA [100]
DOCK8	AGTGCCGAGGACTTTGAGAA/ ATTCTGTTGCCCAGGTGTTC
EGR1	TGACCGCAGAGTCTTTTCCT/ TGGGTTGGTCATGCTCACTA
NEK11	AGAGGATGCCACATCTGACC/ GAAGTGCAACCCAGGACATT
ZNF676	CTGGTCTTCCTGGGTATTGC/ TTGCTCTGGCCAAAACTCTT
CA9	TAAGCAGCTCCACACCCTCT/ TCTCATCTGCACAAGGAACG
COL19A1	GTGGTTTCTGTGGCAGGTTT/AGTCTGCCTCCTCGCAATTA
DACH1	GTGGAAAACACCCCTCAGAA/ CTTGTTCCACATTGCACACC
EGR2	CCTCCTTATTCTGGCTGTGC// CTGGGATCATTGGGAAGAGA
FAP	CTTGTCCTGGCTTCAGCTTC/ AGGTGGCAACTCCAAATACG
HS6ST3	GGCTCACTGAGTTCCAGAGG/ TCTAGCTGCTTGGTGTGGTG
IL2RB	GCTGATCAACTGCAGGAACA/ TGTCCCTCTCCAGCACTTCT
PIP5KIB	CCAGGAATGGAAGGATGAGA/ AATTGTGGTTGCCAAGGAAG
SERPINA3	CCAACGTGGACTTCGCTTTC/CTCTTGGCATCCTCCGTGAA
SERPINB4	TCAGTGAAGCCAACACCAAG/ TGTTGCAGCTTTTTCTGTGG
TNRFS9	CACTCTGTTGCTGGTCCTCA/ CACAGGTCCTTTGTCCACCT
VCAM1	CAGACAGGAAGTCCCTGGAA/ TTCTTGCAGCTTTGTGGATG
ADAMTS5	CCCAGCCTGGACACATTACT/ TTCCCCTGAGCATTTTTCAC
AREG	TGGATTGGACCTCAATGACA/ AGCCAGGTATTTGTGGTTCG
CDK20	ATGGCTAAGGTGGCATTGTC/ CGCTCATCCTGAGGGAGTAG
COL1A2	CCTGGTAATCCTGGAGCAAA/ TTACCGCTCTCTCCTTTGGA
CXCL1	AGGGAATTCACCCCAAGAAC/ CACCAGTGAGCTTCCTCCTC
ITGA8	CACATTCTGGTGGACTGTGG/ AATCCCTTGTTGTTGCGTTC
MMP10	GGCTCTTTCACTCAGCCAAC/ GGCTCTTTCACTCAGCCAAC
PCDH18	AGCATCTGCAGCTTTTCCAT/ AGGGAATTTTCCCCAACATC
SULT1B1	GGTTATCCCATGACCTGTGC/CCAGGGAGAGTCATTTCCAA

#### Nuclear extraction and transcription-factor array analysis

We prepared nuclear extracts from FLs1 or NLSdel cells, which contained activated transcription factors related to cell proliferation. Extracts were prepared with the Active Motif nuclear extraction kit (Rixensart, Belgium, cat. no. 40010). Cells were collected and resuspended in hypotonic buffer, which contained detergents. The cytoplasmic fraction was removed, and cell nuclei were lysed and solubilized in a lysis buffer, which contained protease inhibitors and 10 mM DTT. The protein concentrations in nuclear extracts were measured with the bicinchoninic acid (BCA) assay (Thermo Scientific, IL, USA, cat. no. 23225) at an optical density of 562 nm. The activity of cell growth-related transcription factors was profiled with TranSignal™ Cell Growth Protein/DNA Arrays (Affymetrix Inc., Panomics). 3 μg of nuclear extracts were preincubated with a set of biotin-labeled DNA binding oligonucleotides (TranSignal Probe Mix) to allow the formation of DNA/protein complexes; then, the protein/DNA complexes were separated from the free probes with spin column separation. The probes in the complexes were extracted and hybridized to the TranSignal Array membrane in an overnight incubation at 42 °C. The array was spotted (in duplicate, and at two dilutions) with consensus sequences that corresponded to 20 different transcription factors, which were known key players in cell growth and differentiation. We detected the hybridized signals with HRP-based chemiluminescence detection. The membranes were exposed to a chemiluminescence imaging system (FluorChem™ SP, Alpha Innotech, USA) for 5–10 min. Different signals corresponded to differently activated transcription factors from the nuclear extracts. Results were quantified with the ImageJ 1.47, open-source image analysis program. We calculated the ratio of data collected from FLs1 cells versus those collected from NLSdel cells. Three independent experiments were performed. The threshold for significance was a 1.5-fold change for each experiment.

## Additional files

Additional file 1: Figure S1. Syndecan-1 protein level following transfection with the full-length syndecan-1 (FLs1), nuclear localization signal deleted syndecan-1 (NLSdel) and empty vector control (EV). (**a**) Representative histogram of syndecan-1 protein level detected by Fluorescence Activated Cell Sorting (FACS) analysis. Dotted line represents the IgG control, green line corresponds to empty vector and the blue and red line to the full-length syndecan-1 (FLs1) and nuclear localization signal deleted syndecan-1 (NLSdel), respectively. (**b**) Quantitative syndecan-1 protein level by FACS analysis corresponding to three independent experiments. Error bars represent standard error of the mean (SEM). * denotes statistically significant differences. (**c**) Relative syndecan-1 levels measured by western blotting, using actin as loading control. (JPEG 175 kb)

Additional file 2: Figure S2. Immunocytochemical staining and subcellular localization of the newly synthesized syndecan-1. Panels (**a**, **d** and **g**) represent empty vector, (b, e and g) represent the nuclear localization signal deleted syndecan-1 (NLSdel) and panels (c, f and i) represent full-length syndecan-1 (FLs1) transfected cells. Green staining (a-c) shows syndecan-1, blue color shows (d-f) nuclear staining (Bisbenzimide H33342). Panels (g-i) show overlay of syndecan-1 and the nuclear staining. Immunoreactivity for syndecan-1 is observed mainly in the cell membrane and cytoplasm. In FLs1 syndecan-1 is localized also in the cell nucleus. The amount of total syndecan-1 is lower in empty vector than in the other two constructs. (TIFF 38 kb)

Additional file 3: Figure S3. Ki-67 proliferation index of the full length syndecan-1 (FLs1); nuclear localization signal deleted syndecan-1 (NLSdel); and Empty vector control (EV). Black bars represent the proportion of Ki-67 positive cells at 48 and gray bars at 72 h, respectively. (TIFF 624 kb)

Additional file 4: Figure S4. (**a**) At the level of the global proteome, the amplitudes of changes are small; less than 0.5% of the proteins showed >1.5-fold changes in regulation for each replicate. (**b**) Clustering of one minus the Pearson coefficient, in both columns (samples/replicates) and rows (proteins), shows that two of the replicates had patterns distinct from their respective groups (FL rep3 and NLSdel rep1). However, common features can be discerned between the remaining samples in the groups. (TIFF 523 kb)

Additional file 5: Figure S5. Moderated F-test results show proteins that are significantly regulated (Benjamini-Hochberg corrected *p*-value <0.05; red dots) between the full-length syndecan-1 group (FL) and the group with a syndecan-1 that lacked the nuclear localization signal (NLSdel). Numbers represent Pearson r correlations. The replicates, FL rep3 and NLSdel rep1, show discrepancies in protein expression. However, the other samples show good correlations (*r* > 0.50). (JPEG 748 kb)

Additional file 6: File S1. Differentially regulated proteins between the FLs1 and the NLSdel groups Sheet: “All samples” regards the two sample moderated t-test using all samples in each group. Sheet: “2 vs 2 samples” regards the same two sample moderated t-test analysis but excluding two replicates with low Pearson correlation. id = Uniprot accession numbers (XLSX 1981 kb)

Additional file 7: File S2. List of pathways enriched following nuclear translocation of syndecan-1, identified by GSEA analysis The GSEA analysis performed on the transcriptomic dataset of NLSdel versus Fls1 (sheet “mRNA”) and the proteome dataset (sheet “Proteome”) identified several enriched pathways. (XLSX 141 kb)

## Abbreviations

DOCK8: dedicator of cytokinesis 8
EGR-1: early growth response 1
FLs1: cells transfected with full-length syndecan-1
GO: Gene ontology
HS: heparan sulfate
HSPG: heparan sulfate proteoglycan
NEK11: never-in-mitosis gene a-related kinase 11
NLS: nuclear localization signal
NLSdel: cells transfected with syndecan-1 lacking the nuclear localization signal
V: cells transfected with empty vector
