# Supplementary figures and images for: Molecular targets and signaling pathways regulated by nuclear translocation of syndecan-1
Source: BMC Cell Biol. 2017 Dec 8;18:34. doi: 10.1186/s12860-017-0150-z (PMC5721467; doi:10.1186/s12860-017-0150-z)

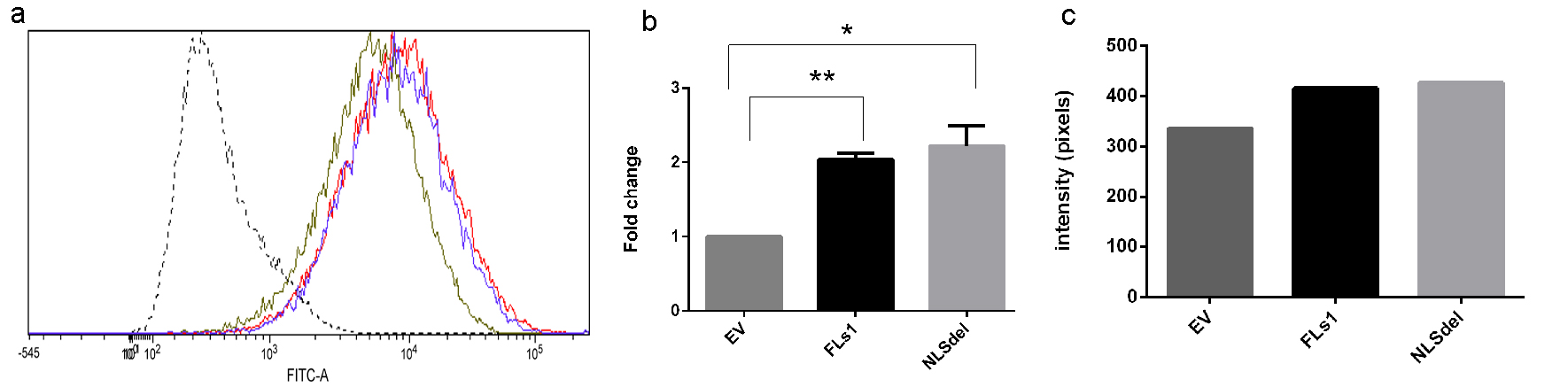

Supplement: Supplementary file 1 — Syndecan-1 protein level following transfection with the full-length syndecan-1 (FLs1), nuclear localization signal deleted syndecan-1 (NLSdel) and empty vector control (EV). (a) Representative histogram of syndecan-1 protein level detected by Fluorescence Activated Cell Sorting (FACS) analysis. Dotted line represents the IgG control, green line corresponds to empty vector and the blue and red line to the full-length syndecan-1 (FLs1) and nuclear localization signal deleted syndecan-1 (NLSdel), respectively. (b) Quantitative syndecan-1 protein level by FACS analysis corresponding to three independent experiments. Error bars represent standard error of the mean (SEM). * denotes statistically significant differences. (c) Relative syndecan-1 levels measured by western blotting, using actin as loading control. (JPEG 175 kb) [file 12860_2017_150_MOESM1_ESM.jpg]

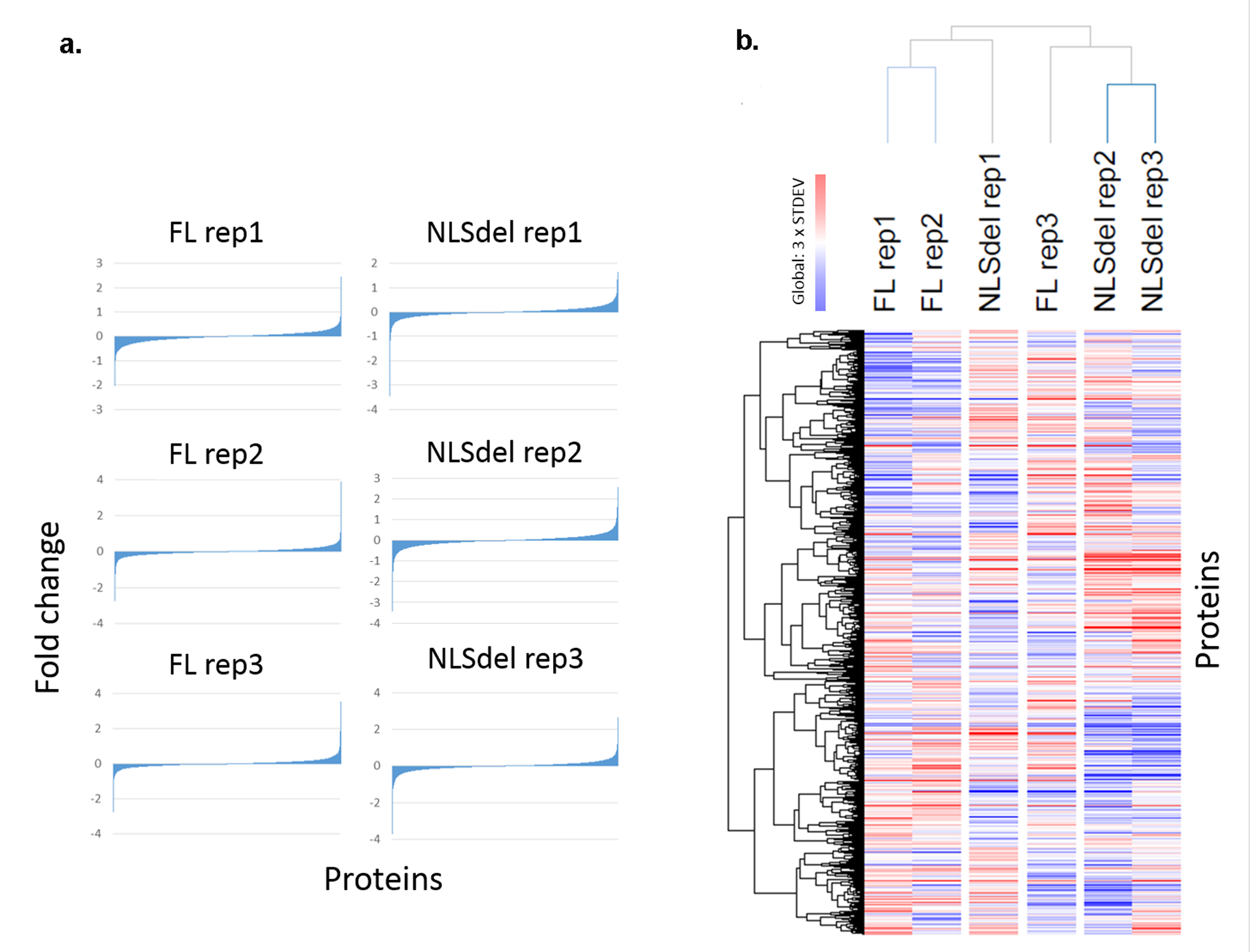

Supplement: Supplementary file 3 — Ki-67 proliferation index of the full length syndecan-1 (FLs1); nuclear localization signal deleted syndecan-1 (NLSdel); and Empty vector control (EV). Black bars represent the proportion of Ki-67 positive cells at 48 and gray bars at 72 h, respectively. (TIFF 624 kb) [file 12860_2017_150_MOESM3_ESM.tiff]

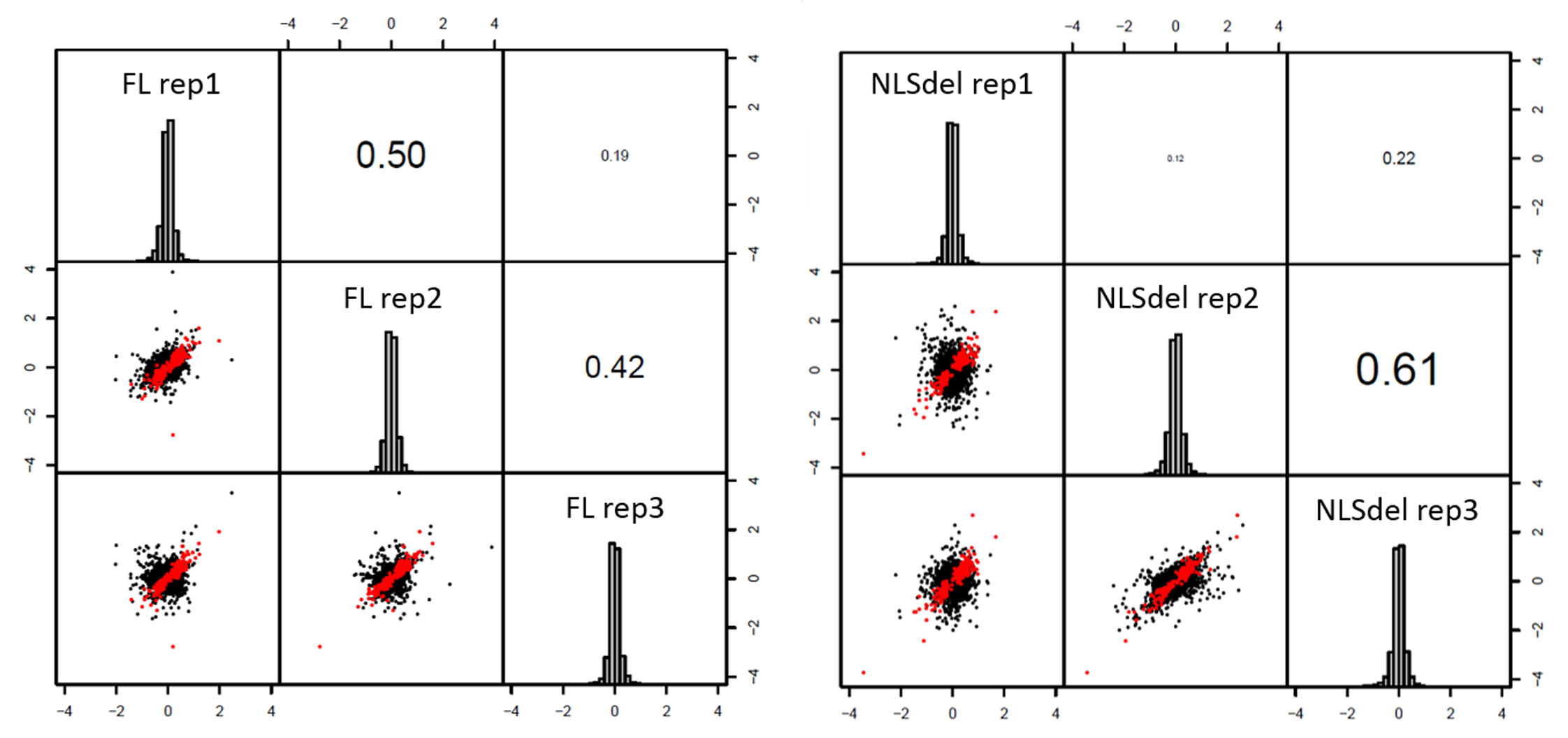

Supplement: Supplementary file 4 — (a) At the level of the global proteome, the amplitudes of changes are small; less than 0.5% of the proteins showed >1.5-fold changes in regulation for each replicate. (b) Clustering of one minus the Pearson coefficient, in both columns (samples/replicates) and rows (proteins), shows that two of the replicates had patterns distinct from their respective groups (FL rep3 and NLSdel rep1). However, common features can be discerned between the remaining samples in the groups. (TIFF 523 kb) [file 12860_2017_150_MOESM4_ESM.tiff]

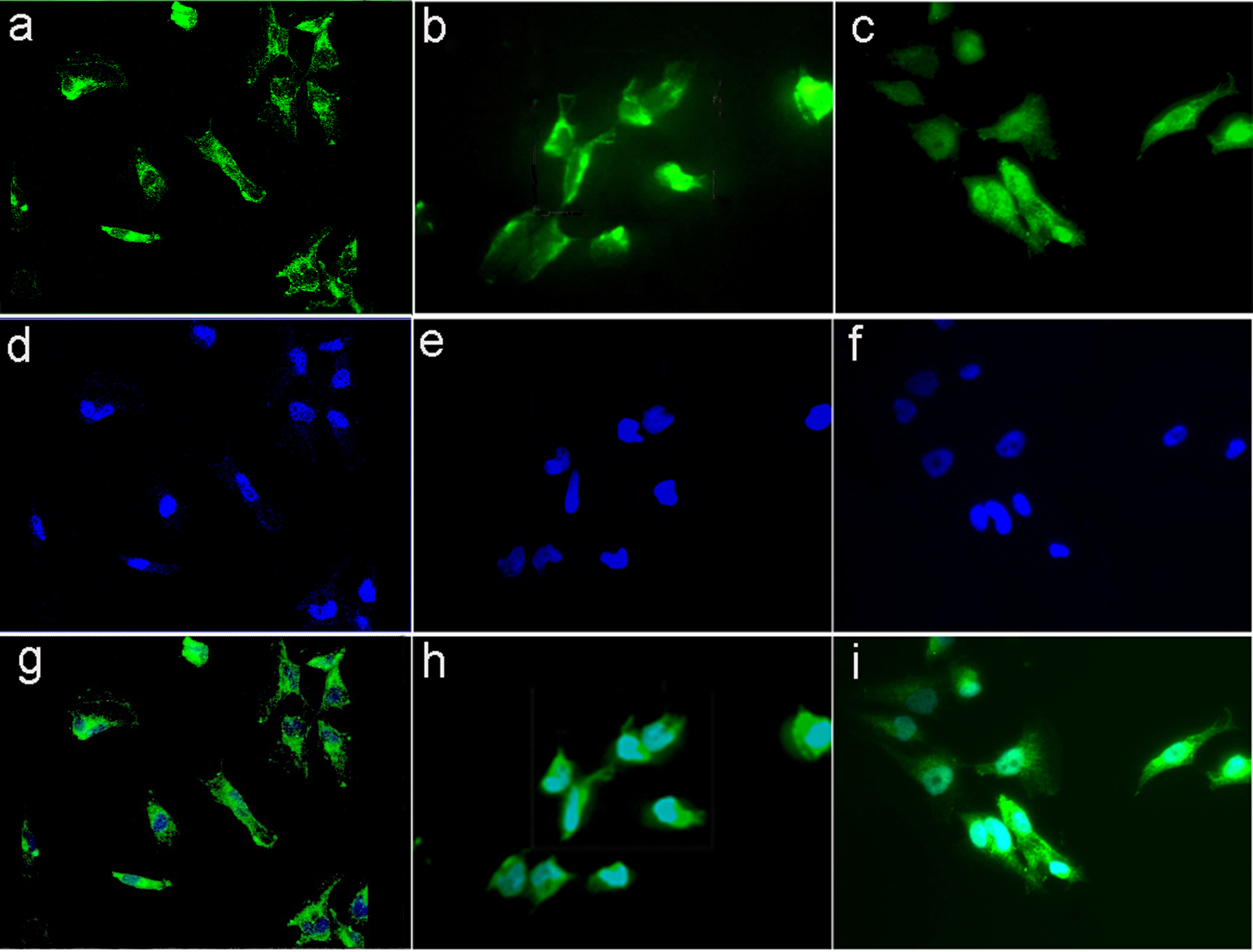

Supplement: Supplementary file 5 — Moderated F-test results show proteins that are significantly regulated (Benjamini-Hochberg corrected p-value <0.05; red dots) between the full-length syndecan-1 group (FL) and the group with a syndecan-1 that lacked the nuclear localization signal (NLSdel). Numbers represent Pearson r correlations. The replicates, FL rep3 and NLSdel rep1, show discrepancies in protein expression. However, the other samples show good correlations (r > 0.50). (JPEG 748 kb) [file 12860_2017_150_MOESM5_ESM.jpg]
